# ^18^F-FDG PET and DCE kinetic modeling and their correlations in primary NSCLC: first voxel-wise correlative analysis of human simultaneous [18F]FDG PET-MRI data

**DOI:** 10.1186/s13550-020-00671-9

**Published:** 2020-07-30

**Authors:** Florent L. Besson, Brice Fernandez, Sylvain Faure, Olaf Mercier, Andrei Seferian, Xavier Mignard, Sacha Mussot, Cecile le Pechoux, Caroline Caramella, Angela Botticella, Antonin Levy, Florence Parent, Sophie Bulifon, David Montani, Delphine Mitilian, Elie Fadel, David Planchard, Benjamin Besse, Maria-Rosa Ghigna-Bellinzoni, Claude Comtat, Vincent Lebon, Emmanuel Durand

**Affiliations:** 1 Université Paris-Saclay, CEA, CNRS, Inserm, BioMAPs, 91401 Orsay, France; 2 Department of Biophysics and Nuclear Medicine-Molecular Imaging, Hôpitaux Universitaires Paris-Saclay, Assistance Publique-Hôpitaux de Paris, CHU Bicêtre, 94270 Le Kremlin-Bicêtre, France; 3 School of Medicine, Université Paris-Saclay, Le Kremlin-Bicêtre, France; 4 Applications and Workflow, GE Healthcare, Orsay, France; 5 Laboratoire de Mathématiques d’Orsay, CNRS, Université Paris-Saclay, 91405 Orsay, France; 6 Department of Thoracic and Vascular Surgery and Heart-Lung Transplantation, Marie Lannelongue Hospital, 92350 Le Plessis Robinson, France; 7 Service de Pneumologie, Centre de Référence de l’Hypertension Pulmonaire, Hôpitaux Universitaires Paris-Saclay, Assistance Publique-Hôpitaux de Paris, 94270 Le Kremlin-Bicêtre, France; 8 Inserm UMR_S999, Marie Lannelongue Hospital, 92350 Le Plessis Robinson, France; 9 Department of Radiation Oncology, Institut d’Oncologie Thoracique (IOT), Gustave Roussy, Université Paris Saclay, Villejuif, France; 10 Department of Radiology, Institut d’Oncologie Thoracique (IOT), Gustave Roussy, Université Paris Saclay, Villejuif, France; 11 Department of Oncology, Institut d’Oncologie Thoracique (IOT), Gustave Roussy, Université Paris Saclay, Villejuif, France; 12 Department of Pathology, Marie Lannelongue Hospital, 92350 Le Plessis Robinson, France

**Keywords:** PET-MRI, DCE-MRI, [18F]FDG, Kinetic parameters, NSCLC, Quantification

## Abstract

**Objectives:**

To decipher the correlations between PET and DCE kinetic parameters in non-small-cell lung cancer (NSCLC), by using voxel-wise analysis of dynamic simultaneous [18F]FDG PET-MRI.

**Material and methods:**

Fourteen treatment-naïve patients with biopsy-proven NSCLC prospectively underwent a 1-h dynamic [18F]FDG thoracic PET-MRI scan including DCE. The PET and DCE data were normalized to their corresponding T_1_-weighted MR morphological space, and tumors were masked semi-automatically. Voxel-wise parametric maps of PET and DCE kinetic parameters were computed by fitting the dynamic PET and DCE tumor data to the Sokoloff and Extended Tofts models respectively, by using in-house developed procedures. Curve-fitting errors were assessed by computing the relative root mean square error (rRMSE) of the estimated PET and DCE signals at the voxel level. For each tumor, Spearman correlation coefficients (*r*_s_) between all the pairs of PET and DCE kinetic parameters were estimated on a voxel-wise basis, along with their respective bootstrapped 95% confidence intervals (*n* = 1000 iterations).

**Results:**

Curve-fitting metrics provided fit errors under 20% for almost 90% of the PET voxels (median rRMSE = 10.3, interquartile ranges IQR = 8.1; 14.3), whereas 73.3% of the DCE voxels showed fit errors under 45% (median rRMSE = 31.8%, IQR = 22.4; 46.6). The PET-PET, DCE-DCE, and PET-DCE voxel-wise correlations varied according to individual tumor behaviors. Beyond this wide variability, the PET-PET and DCE-DCE correlations were mainly high (absolute *r*_s_ values > 0.7), whereas the PET-DCE correlations were mainly low to moderate (absolute *r*_s_ values < 0.7). Half the tumors showed a hypometabolism with low perfused/vascularized profile, a hallmark of hypoxia, and tumor aggressiveness.

**Conclusion:**

A dynamic “one-stop shop” procedure applied to NSCLC is technically feasible in clinical practice. PET and DCE kinetic parameters assessed simultaneously are not highly correlated in NSCLC, and these correlations showed a wide variability among tumors and patients. These results tend to suggest that PET and DCE kinetic parameters might provide complementary information. In the future, this might make PET-MRI a unique tool to characterize the individual tumor biological behavior in NSCLC.

## Introduction

Positron emission tomography (PET) combined with magnetic resonance imaging (MRI) emerged a decade ago [1, 2]. Since then, substantial efforts have been made to promote its clinical use, but disappointing results compared to more cost-effective and former imaging modalities still make the positioning of PET-MRI challenging in clinical practice [3]. In the era of precision medicine, advanced multiparametric imaging offers many opportunities to better characterize the biological processes of tumors [4–6]. In contrast to standard visual or semi-quantitative imaging methods, the more advanced dynamic quantitative imaging approach allows the absolute quantification of various sophisticated biological processes, based on the pharmacokinetic modeling of tracer exchanges. PET kinetic modeling of [18F]FDG quantifies the glucose metabolic pathway in tumor cells, characterized by the related K_1_, k_2_, and k_3_ PET microparameters [7]. Previous dynamic PET studies showed [18F]FDG microparameters to be surrogates of tumor aggressiveness or prognosis factors in a wide variety of malignancies [8–11], including primary non-small-cell lung cancer model (NSCLC) [12]. In magnetic resonance imaging (MRI), dynamic contrast-enhanced MRI (DCE-MRI) provides insight into the underlying tumor vascularization at the microcirculatory level, depending on the contrast agent leakage through the capillary wall. In the case of gadolinium (Gd), the K_trans_, v_e_, K_ep_ (the K_trans_ to v_e_ ratio), and v_p_ microparameters reflect the perfusion, permeability, and microvascular density properties of the tumor [13]. Previous oncological studies showed DCE microparameters to be significant predictors of response to treatment in several malignancies [14–17], including NSCLC [18].

Metabolism and vascularization are two fundamental hallmarks of cancer [19], and their relationships are of particular relevance to capture the tumor progression and responses to treatment capabilities [20]. In integrated PET-MRI, combining PET and DCE kinetic modeling may be thus of particular interest to revisit the complex relationship between these two fundamental tumor hallmarks [19, 20] at the intra-tumor regional level. In lung cancer, previous [18F]FDG PET/MRI imaging studies have been performed mainly for clinical disease staging evaluation [21–25], SUV-ADC correlation analyses [26–29], and prognostic value [30]. To date, only a few multimodal imaging studies compared tumor metabolism assessed with PET and angiogenesis assessed with DCE in NSCLC [31–35], of which only two combined dedicated [18F]FDG PET and DCE-MRI imaging data [31, 35]. So far, a combined voxel-wise analysis of simultaneous dynamic [18F]FDG PET and DCE-MRI has never been performed at the individual tumor level.

In this study, we deciphered the correlations between [18F]FDG PET and DCE kinetic parameters at the intra-tumor level in newly diagnosed, biopsy proven NSCLC, by using a combined voxel-wise analysis of dynamic simultaneous [18F]FDG PET-MRI.

## Material and methods

### Patients

Between January 2018 and April 2019, a total of 14 treatment-naïve patients with biopsy proven NSCLC prospectively underwent a dynamic [18F]FDG PET-MRI for thoracic oncology purposes. The exclusion criteria were claustrophobia, metal implants, renal failure (clearance < 30 mL/min), and uncontrolled diabetes mellitus. Patient characteristics are summarized in Table 1. The local institutional review board approved this study (SHFJ Research Steering Committee, DRF/JOLIOT/SHFJ/2020/10), and all patients signed written informed consent.

**Table 1:** Patients characteristics

Patient	Age	Gender	NSCLC localization	Histology	Voxels (2 mm^3^)
1	82	M	Right upper lobe	Poorly differentiated NSCLC	540
2	47	M	Right upper lobe	NSCLC	1271
3	71	F	Right lower lobe	NSCLC (undifferentiated carcinoma)	799
4	67	F	Left upper lobe	NSCLC (ADK)	211
5	80	F	Left upper lobe	NSCLC (SCC)	1207
6	53	M	Right medium lobe	NSCLC (ADK)	88
7	78	F	Left upper lobe	NSCLC (ADK)	629
8	55	M	Right upper lobe	NSCLC (ADK)	318
9	63	M	Left upper lobe	NSCLC (poorly differentiated SCC)	2409
10	57	M	Left upper lobe	NSCLC (ADK)	2338
11	62	M	Right upper lobe	NSCLC (ADK)	1151
12	61	M	Right upper lobe	NSCLC (SCC)	5340
13	71	F	Right upper lobe	NSCLC (ADK)	3845
14	71	M	Right upper lobe	NSCLC (ADK)	1409

*ADK* adenocarcinoma, *SCC* squamous cell carcinoma

### PET/MRI

All the examinations were performed in the supine position on the same integrated 3 T PET-MRI scanner (Signa PET/MR, GE Healthcare, Waukesha, WI, USA). All patients fulfilled the international procedure guideline for [18F]FDG PET tumor imaging [36], having fasted for 6 h and a blood glucose level under 1.8 g/L at the time of the imaging procedure. A 1-h dynamic thoracic PET acquisition started immediately after the intravenous injection of 3–4 MBq/kg of [18F]FDG. The dynamic PET data were histogrammed into multiframe sinograms (41 frames of 12 × 10 s, 12 × 20 s, 4 × 60 s, 5 × 120 s, 8 × 300 s, respectively) to be reconstructed using an iterative algorithm (3D TOF-OSEM, 6 iterations, and 28 subsets with time of flight and point spread function modeling, and with random, dead time, scatter, decay, and attenuation corrections, matrix size = 256 × 256; voxel size = 2 × 2 × 2.78 mm). Simultaneously, the following MR thoracic acquisitions were performed using a thoracic phased array radiofrequency (RF) coil (GEM Coil Suite, GE Healthcare, Waukesha, WI, USA):

- A two-point (fat, water) axial 3D-Dixon pulse sequence (TR/TE_1_/TE_2_ = 4/1.1/2.2 ms, Field-Of-View (FOV): 500/500/332.8 mm, number of excitations (NEX) = 0.7, voxel size 1.95 × 1.95 × 2.6 mm) for MR-based attenuation correction.
- A PROPELLER fast recovery Fast Spin Echo sequence with respiratory triggering for T2-weighted morphology (TR/TE = 8000/117 ms, FOV: 400/400/90 mm, NEX = 2; voxel size 1.0 × 1.0 × 6.0 mm, acceleration factor = 3).
- A 2D saturation recovery pulse sequence for pre-contrast T_1_-mapping (cardiac triggered, inversion times = [136/136/136/136/818/1583/2109/2808/20000] ms, TR/TE = 2.9/1.1 ms; FOV: 420/420/30 mm, NEX = 1; voxel size 1.64 × 1.64 × 5.0 mm) [37].
- DCE acquisitions performed before, during, and after the automated injection of gadolinium contrast agent (Gd, 0.2 mmol/kg body weight, Dotarem, Guerbet GmbH, Germany; injecting rate of 2.0 mL/s by power injector) using 3D T_1_-Fast Spoiled Gradient Recalled (Fast SPGR) pulse sequences under free breathing (120 frames of 3.03 s each for a total acquisition time of 6 min, TR/TE = 3.46/1.10 ms; FOV: 400/320/120 mm, NEX = 0.69, voxel size 1.56 × 1.25 × 2.5 mm).
- A post-contrast 3D T_1_-Fast SPGR sequence in breath-hold position (TR/TE = 4.48/2.41 ms, FOV: 440/352/179.2 mm, NEX = 0.7, voxel size 1.72 × 1.72 × 0.8 mm).

### Image processing

**Fig. 1:**
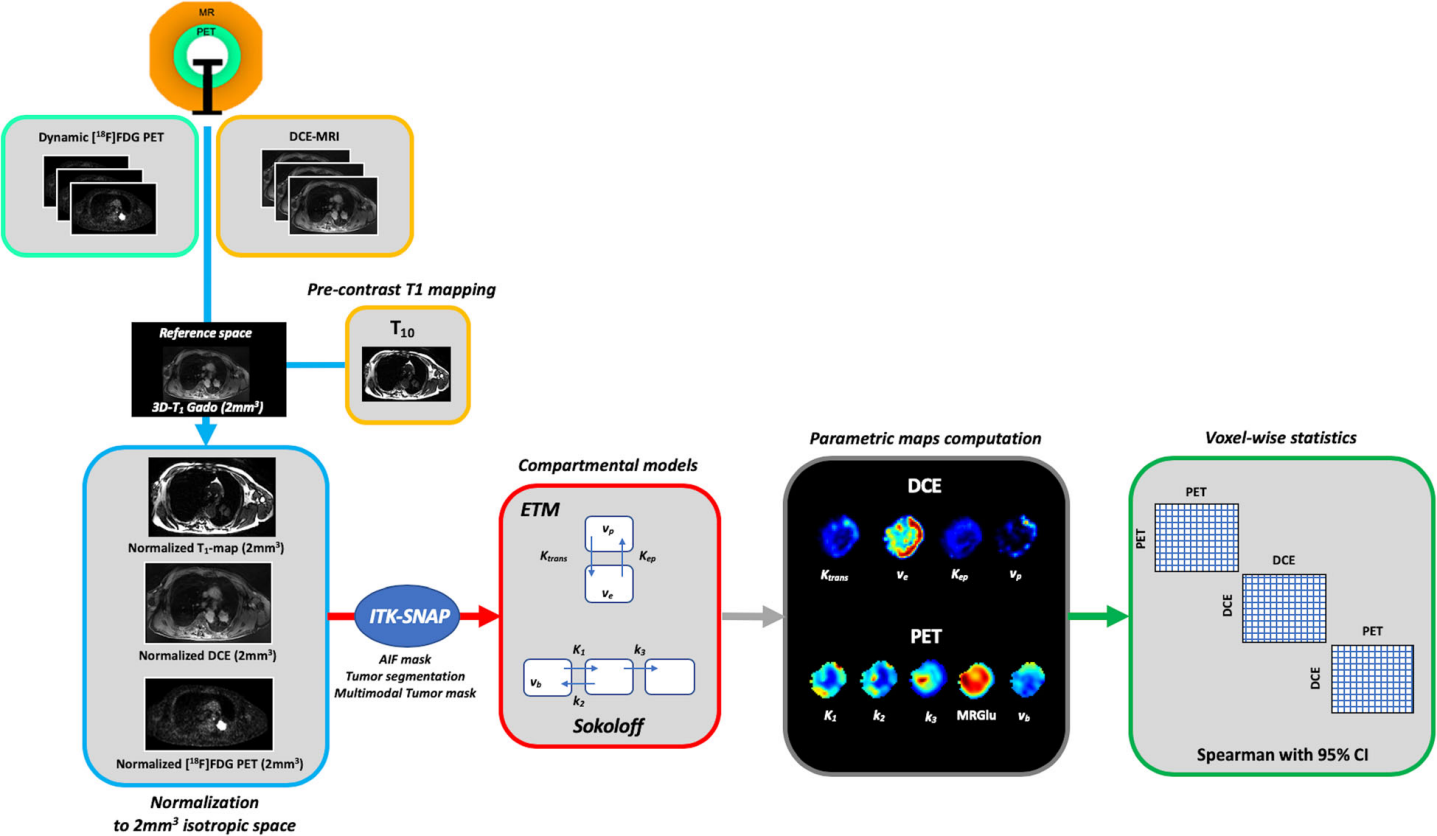
Study workflow. ETM, extended Tofts model

Because no software or dedicated professional workstation currently allows the multimodal voxel-wise computation of PET and DCE parametric maps in PET-MRI, all data processing was performed on a stand-alone personal computer using in-house developed software written in Python (version 3.6; Python Software Foundation, www.python.org; libraries numpy, pandas, nibabel, nilearn, nipype, scipy, math). The general study workflow is provided in the Fig. 1. For each patient, the same image processing was performed as follows:

- Data normalization: [18F]FDG-PET and DCE-MRI data were first normalized to the 3D-T_1_ reference isotropic space (i.e., the post-contrast 3D T_1_-weighted MRI resampled to 2 mm^3^ isotropic). For this purpose, the dynamic PET data and the MR pre-contrast T_1_-mapping data were resampled to the 3D-T_1_ space (libraries nilearn and nibabel), whereas the DCE data were motion-compensated (warping to the 3D-T_1_ space) using the SyNQuicK procedure (library nipype, defaut parameters) implemented in Advanced Normalization Tools (ANTs) [38, 39].
- Tumor mask: the last frame of [18F]FDG-PET and DCE data, the pre-contrast T_1_-mapping data, and the post-contrast 3D-T_1_ data were masked semi-automatically with ITK-SNAP (http://www.itksnap.org), which implements an active contour-based algorithm [40, 41], as follows: an intensity-grading feature image was first computed to define the lesion boundaries by thresholding the intensities of the input image into the background and foreground (region competition approach, in which the intensity values ranged from − 1 to 1 for background and foreground respectively); one or more spherical seeds were then placed on the feature image to initialize the segmentation task; and the iterative algorithm was launched to propagate the seeds, driven by regularity constraints and the image intensity properties. The resulting PET, DCE, T_1_-mapping, and 3D-T_1_ tumor masks were combined into a single multimodal tumor mask (library nilearn) using a basic intersection operation.
- Arterial mask for image-based derived input function (IDIF): IDIF is non-invasive and has been validated against arterial sampling (the gold standard) in oncological patients [42]. IDIF was performed with the graphical user interface ITK-SNAP as follows: a small volume of interest (VOI) was carefully positioned on the center of the thoracic aorta to avoid spill-in and spill-over effects. The position was carefully chosen to fit within the FOVs of all the PET, T_1_-mapping, and DCE fused data.
- Signal processing: the 4D-PET data were smoothed with an 8-mm Gaussian filter (library nilearn), and the DCE imaging data were converted to gadolinium plasma concentration *C*(*t*) (libraries nilearn, numpy, pandas) using the following equation [43]:
  $$ C(t)=\frac{\ 1\ }{\left(1-\mathrm{Hct}\right)} \times \frac{-1}{r_{Gd}\times TR}\times \left[\frac{TR}{T_{10}}+ Ln\ \left(\frac{\left(\frac{S(t)}{S0}\times \frac{1-E}{1-\cos \left(\alpha \right)E}-1\right)}{\frac{S(t)}{S0}\times \cos \left(\alpha \right)\times \frac{1-E}{1-\cos \left(\alpha \right)E}-1}\right)\right], $$

where *S*_0_ and *S*(t) are the signals measured before contrast-enhancement and at time *t* after contrast injection, Hct is the hematocrit level fixed at 0.45 [44], *r*_*Gd*_ = 3.4 s^−1^.mM^−1^ is the relaxivity of Dotarem at 3 T [45], E = ${e}^{- TR/{T}_{10}}$ where *T*_10_ is the estimated pre-contrast *T*_1_ value in the voxel of interest, and *α* is the flip angle of the 3D Fast SPGR pulse sequence, set to 15° in our imaging protocol.

e) Voxel-wise parametric maps computation: tumor and IDIF data were extracted from the masked 4D PET and DCE data, and the [18F]FDG PET (K_1_, k_2_, k_3_, v_b_, MRGlu) and DCE-MRI (K_trans_, v_e_, K_ep_, v_p_) kinetic parameters were finally computed by fitting the extracted data to the reference Sokoloff’s ([18F]FDG PET) [7] and extended Tofts (DCE) [46] compartmental models on a voxel-wise basis, using “in-house” second order Runge-Kutta procedures combined with Levenberg-Marquardt non-linear least-square fitting optimization (libraries numpy, pandas, scipy, math).

### Statistical analysis

All the statistical analyses were performed with Python (version 3.6; Python Software Foundation, www.python.org) and R studio (version 3.4.0; R Project for Statistical Computing, https://rstudio.com).

Curve fitting errors of our in-house PET and DCE kinetic modeling implementation were assessed voxel-wise by computing the relative root mean square errors (Python, libraries numpy, and pandas), defined by $rRMSE=\frac{{\left\Vert signal-\overline{signal}\right\Vert}_2}{{\left\Vert signal\right\Vert}_2}$ where *signal* is the measured signal and $\overline{signal}$ is the estimated signal after the fitting procedure. The PET and DCE kinetic microparameter values are expressed as median± IQR. After data transformation into *z*-score (zero mean and unit variance) and cleaning-up from outliers (*z*-score > 3), the PET-PET, DCE-DCE, and PET-DCE voxel-wise correlations were assessed for each tumor by estimating the related Spearman coefficients (*r*_s_), along with their respective bootstrapped 95% confidence intervals (R studio, RVAideMemoire package, *n* = 1000 iterations). The absolute *r*_s_ estimated values (|*r*_*s*_|) were considered low under 0.4, moderate between 0.4 and 0.7, and high above 0.7 [47].

## Results

**Fig. 2:**
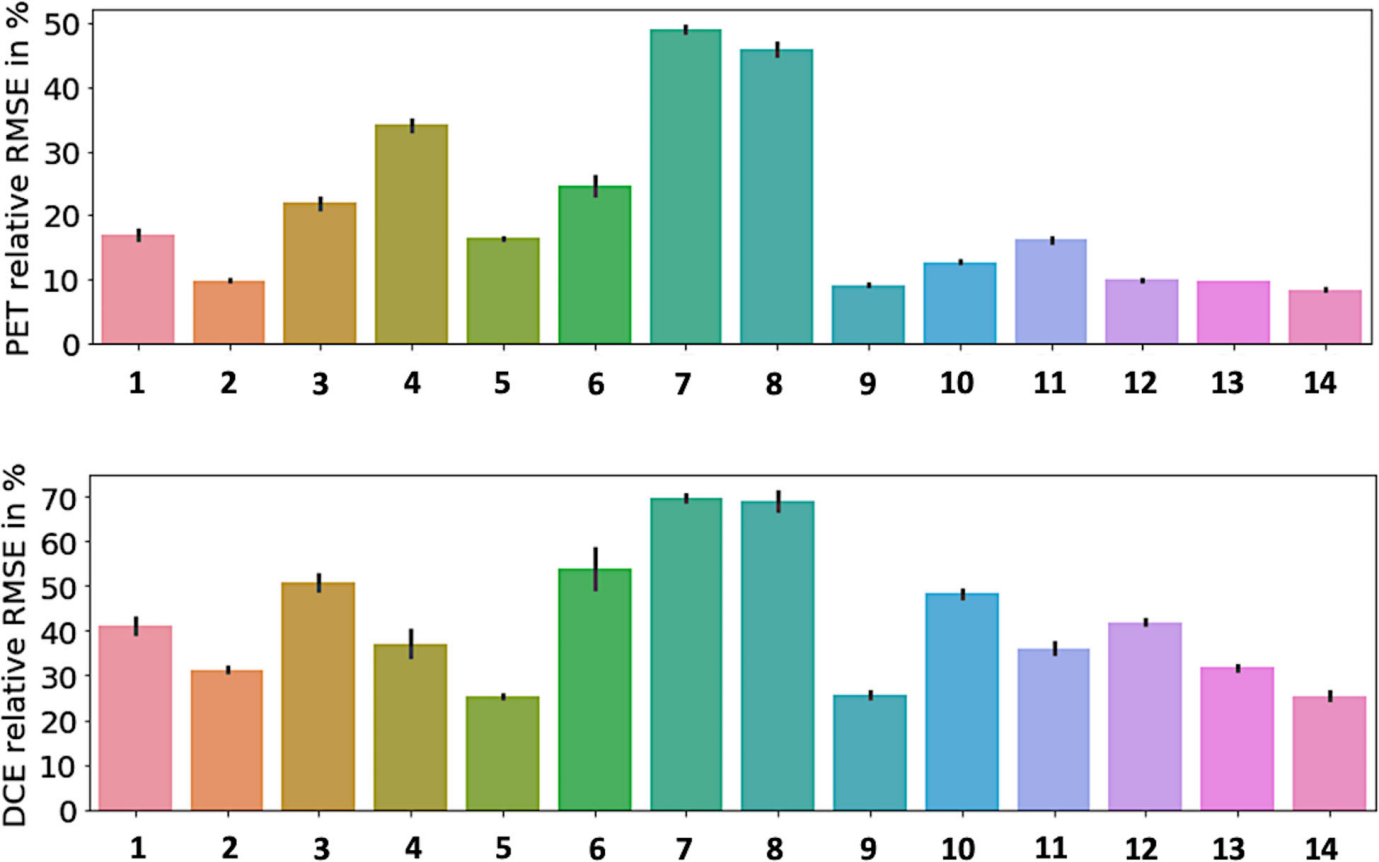
Curve fitting results for PET and DCE kinetic modeling. For each tumor (*x*-axis), voxel-wise relative root mean square errors (relative RMSE) are provided (*y*-axis). For each tumor, the vertical black lines are the standard deviations

**Fig. 3:**
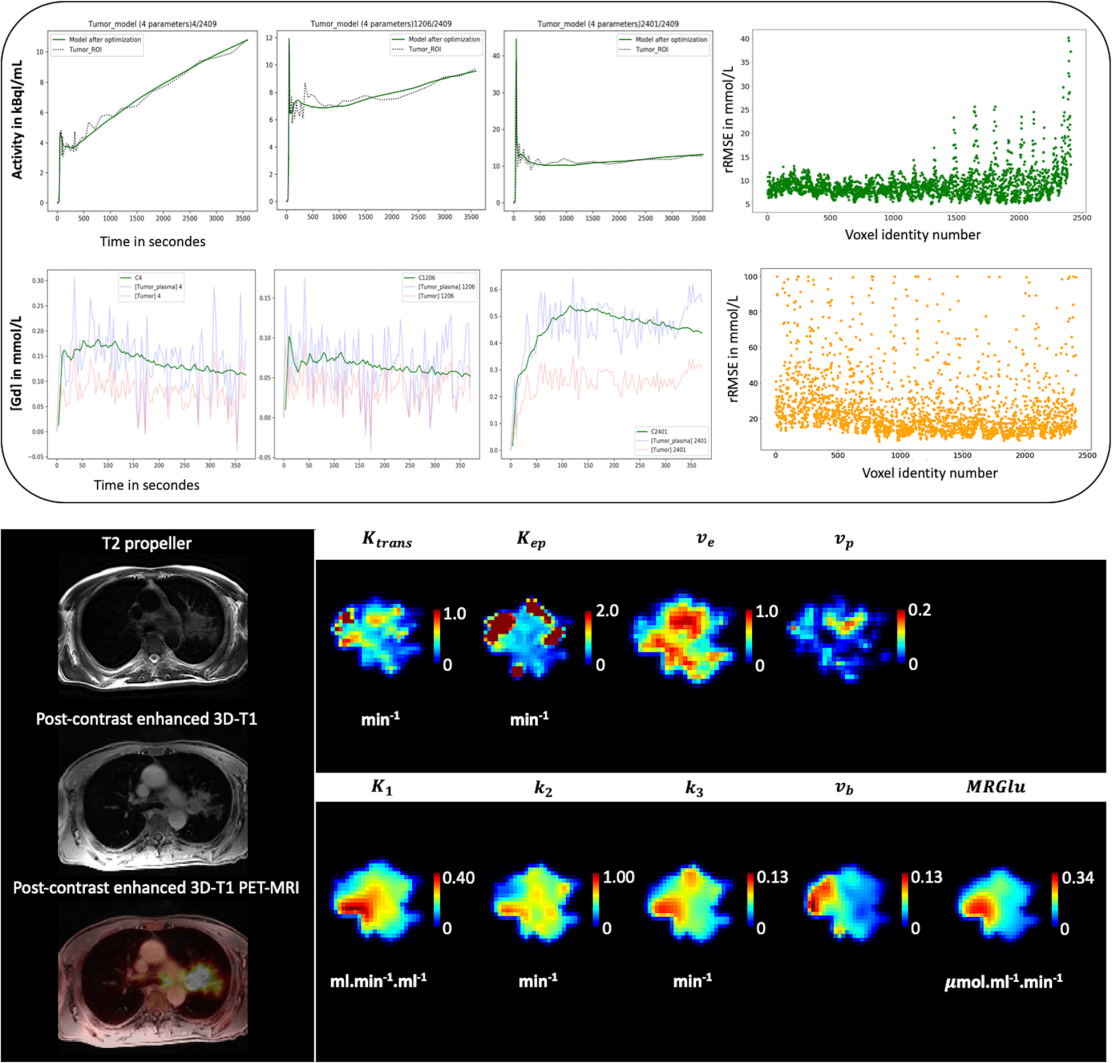
Illustration of the PET and DCE kinetic estimated parameter maps (patient n°9). Top: voxel-wise fitting results are provided for three voxels of interest. The PET signal is expressed in kBq/mL and the DCE signal in mmol/L of Gd. For the latter, the blue curve corresponds to the measured [Gd]_Plasma_, whereas the red one corresponds to the measured [Gd]_Blood_ before plasma conversion. The voxel-wise rRMSE (PET in green, DCE in orange) are also provided at the tumor level. Bottom: the related PET and DCE 3D parametric maps after data fitting

**Fig. 4:**
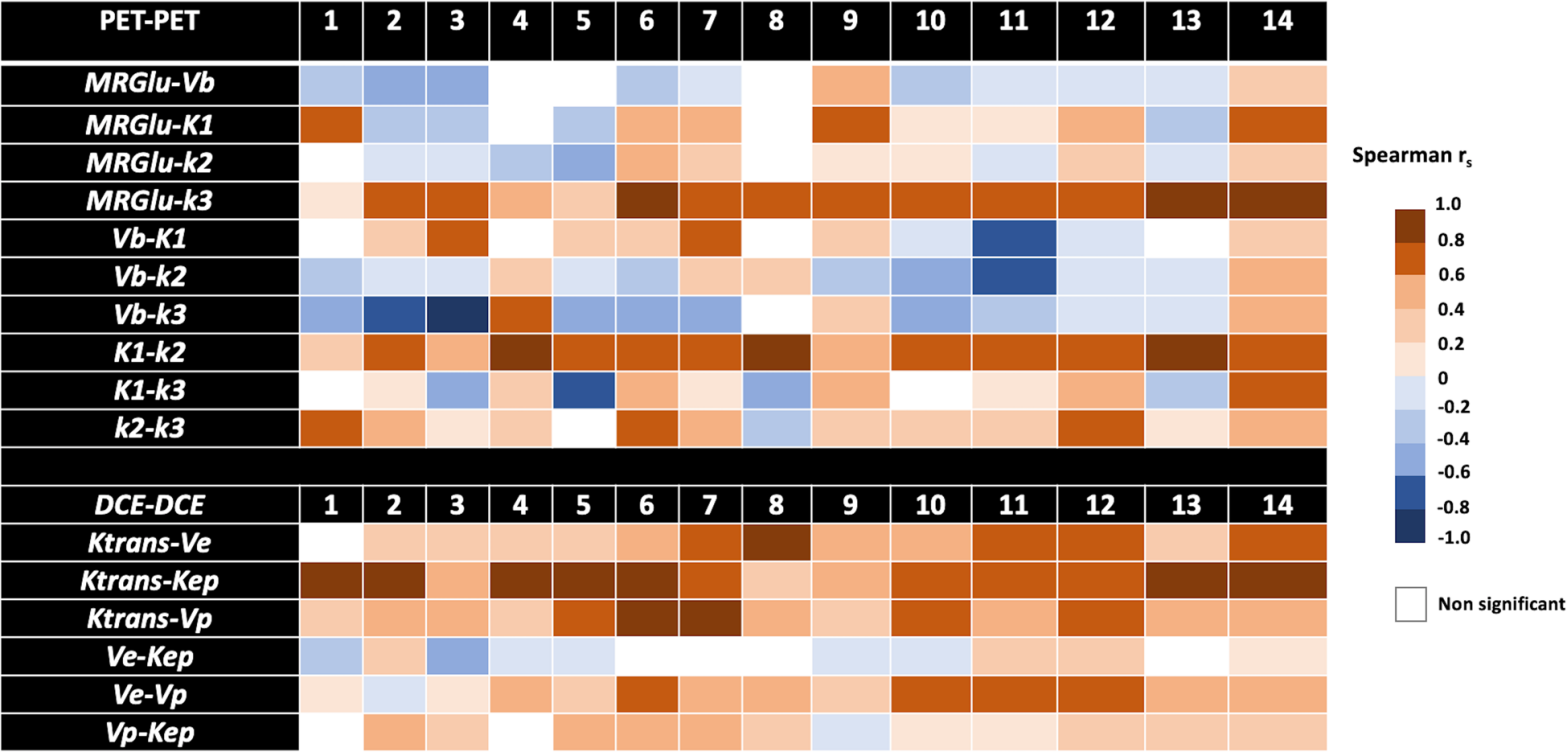
PET-PET and DCE-DCE Spearman correlation. For each tumor (1 to 14), all the PET-PET and DCE-DCE correlation pairs are provided

**Fig. 5:**
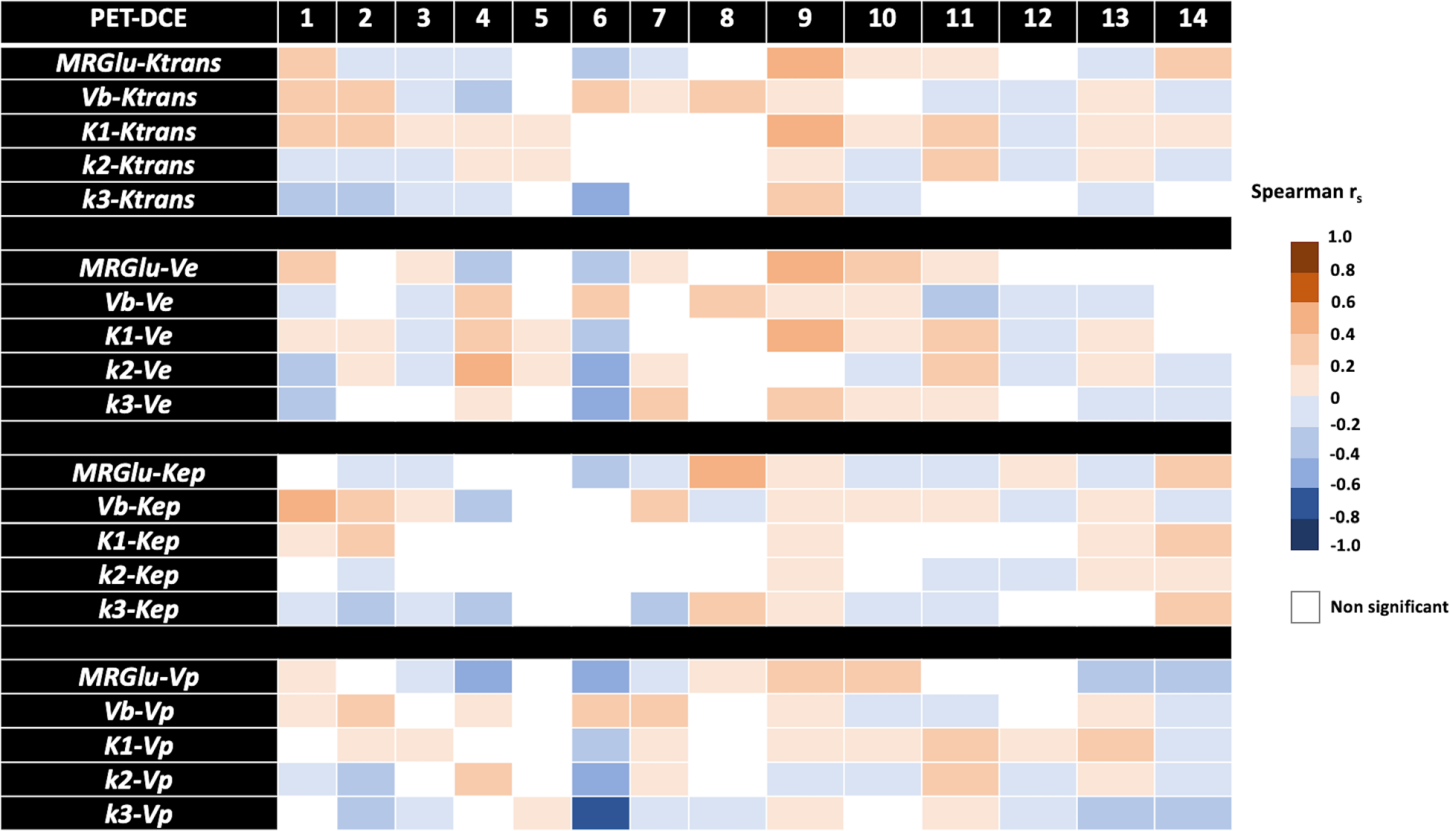
PET-DCE Spearman correlation. For each tumor (1 to 14), all the PET-DCE correlation pairs are provided

**Fig. 6:**
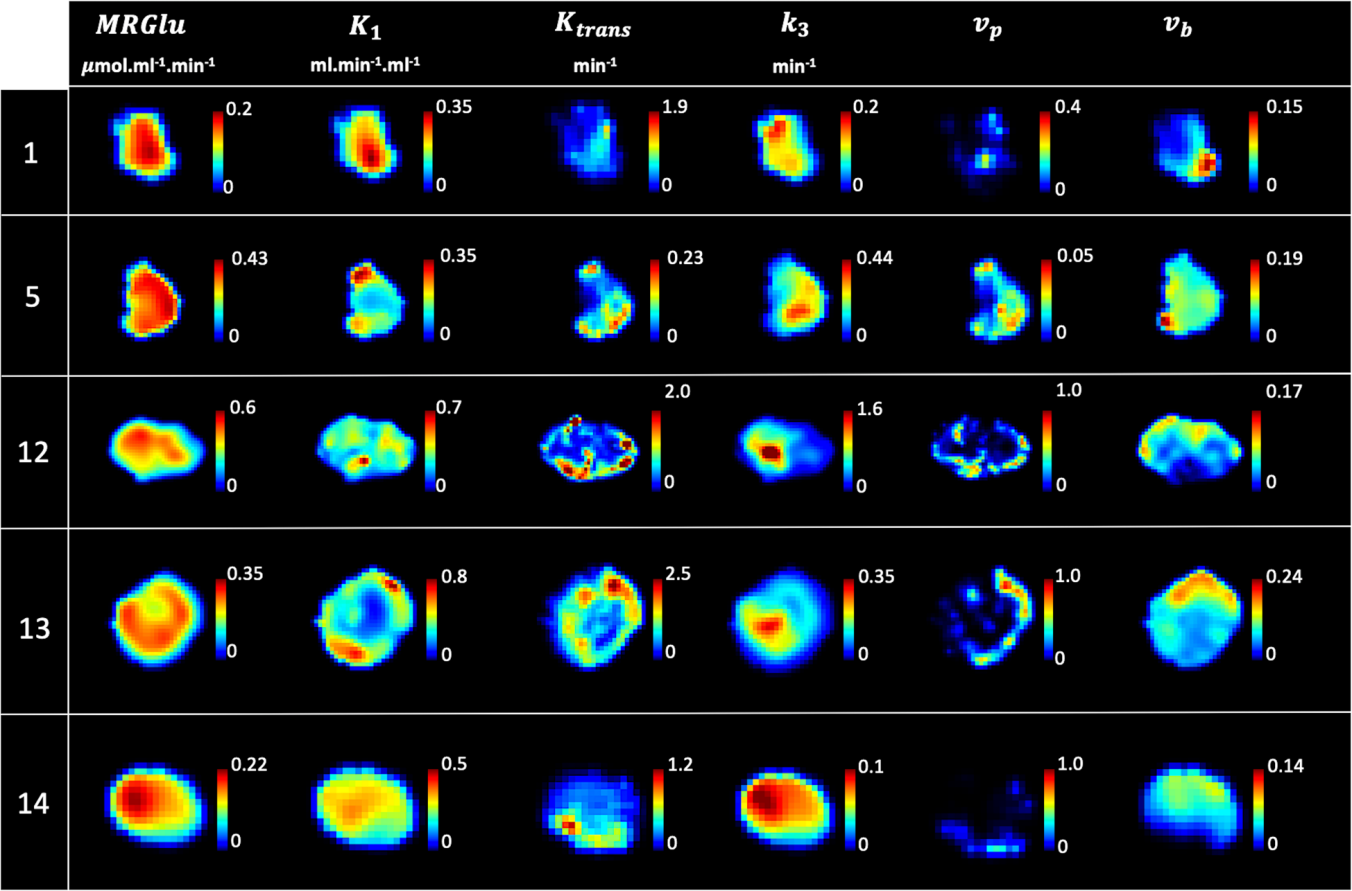
Regional decoupling between perfusion/vascularization and metabolism. In all these tumors, whereas MRGlu appears relatively homogeneous, deep central hypoperfused/vascularized areas of variable sizes are visible (low K_trans_, v_p_, or v_b_), mirrored by high metabolic enzymatic activity (k_3_). This pattern is highly suggestive of hypoxic tumor areas, a well-known hallmark of cancer aggressiveness

The general characteristics of the 14 patients are summarized in Table 1. Briefly, 9 were male, and 5 were female (sex ratio M/F = 1.8), aged 65.5 ± 10.6 years. The tumor localization was the right upper lobe for the majority of the patients (7 patients) or the left upper lobe (5 patients); the two remaining patients had the tumor in the right lower lobe and the right medium lobe, respectively. The estimated [18F]FDG PET and DCE kinetic parameters are summarized in Table 2. The voxel-wise curve-fitting metrics (fit errors) of the PET and DCE kinetic measurements are provided in Tables 3 and 4 and Fig. 2. For the 14 tumors (21,555 estimated voxels), the overall PET rRMSE was 10.3% (8.1; 14.3), corresponding to 89.3% of voxels with error under 20%. The overall DCE rRMSE was 31.8% (22.4; 46.6), corresponding to 73.3% of voxels with error under 45%. An illustration of the PET and DCE kinetic estimated parameter maps of the patient n°9, together with their related curve-fitting statistics, is provided in Fig. 3. The correlation analyses showed wide variability in the PET-PET, DCE-DCE, and PET-DCE correlations (Figs. 4 and 5 and supplementary material). The PET-PET and DCE-DCE correlations were mainly moderate to strong for all the tumors but with high individual variabilities (Fig. 4 and supplementary material). When considering the PET-DCE correlations exclusively, the 14 tumors showed weak (|*r*_s_| < 0.4) to moderate (0.4 ≤  |*r*_s_| <0.7) correlations exclusively (Fig. 5 and supplementary material). MRGlu was positively correlated to k_3_ in all tumors and inversely correlated with K_trans_, v_p_, or v_b_ in the majority of tumors. The 3D parametric maps clearly showed regional decoupling patterns of hypoperfused (K_trans_ or K_1_) and poor vascularized areas (v_b_ or v_p_) with high metabolic enzymatic activity (k_3_) in five tumors, as illustrated in Fig. 6 (tumors 1, 5, 12, 13, and 14).

**Table 2:**
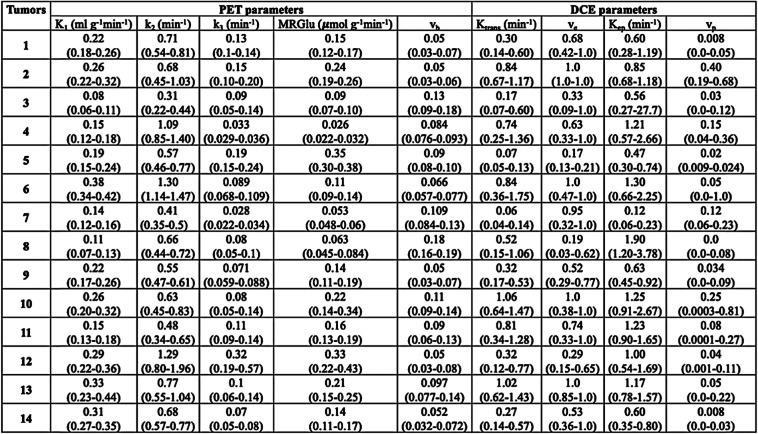
Estimated PET and DCE kinetic parameters. Kinetic parameters are expressed as median (IQR)

**Table 3:** Curve fitting metrics for PET kinetic modeling

PET	Relative RMSE	Fraction of voxels in percent		
		Relative RMSE ≤ 20%	20% < relative RMSE ≤ 45%	45% < Relative RMSE
1	13.4 (10; 20.3)	74%	23%	3%
2	9.0 (6.9; 12.2)	99.6%	0.4%	0%
3	18.1 (12.9; 26.8)	57%	36%	7%
4	34.3 (29; 39)	0%	94.8%	5.2%
5	16.1 (14; 18.1)	87.2%	12.8%	0%
6	23.7 (19.6; 27.3)	27%	72%	1%
7	49.2 (44.1; 54)	0%	29%	71%
8	43.5 (39.7; 51.2)	0%	57.6%	42.4%
9	8.5 (7.4; 9.8)	98.2%	1.8%	0%
10	12.3 (9.8; 15.3)	95.3%	4.7%	0%
11	15.5 (11.5; 19.5)	77.8%	22.2%	0%
12	9.5 (7.6; 11.8)	99.7%	0.3%	0%
13	9.2 (7.7; 11.3)	99.4%	0.6%	0%
14	8.2 (7.4; 9.2)	100%	0%	0%
All	10.3 (8.1; 14.3)	89.3%	7.6%	3.1%

Relative RMSE data are expressed as median (IQR)

**Table 4:** Curve fitting metrics for DCE kinetic modeling

DCE	Relative RMSE	Number of voxels in percent		
		Relative RMSE ≤ 20%	20% < relative RMSE ≤ 45%	45% < relative RMSE
1	35 (26; 51.5)	6.6%	60%	33.4%
2	30.2 (25; 36)	8.3%	87.1%	4.6%
3	46.6 (32.4; 65.3)	3.7%	44%	52.3%
4	29.3 (22.1; 46.9)	21.3%	50%	28.7%
5	23.8 (20.5; 28.5)	21.6%	76%	2.4%
6	49.1 (39.5; 68.5)	1.2%	43%	55.8%
7	68.3 (63.4; 73.2)	0%	0%	100%
8	65.8 (54.9; 84.9)	0%	8.8%	91.2%
9	19.5 (14.2; 29.6)	51.5%	37%	11.5%
10	42.5 (35; 56)	0.1%	56%	43.9%
11	29.8 (23.1; 41.7)	14.5%	64.9%	20.6%
12	36.7 (28; 51.4)	5%	61.4%	33.6%
13	27 (19.7; 40.8)	26.7%	54.2%	19.1%
14	21.1 (15; 29.6)	46.3%	42.5%	11.2%
All	31.8 (22.4; 46.6)	18.6%	54.7%	26.7%

Relative RMSE data are expressed as median (IQR)

## Discussion

This simultaneous dynamic PET-DCE MRI study shows that dynamic PET and DCE monotonic correlations, measured in exactly the same conditions, are highly variable at the tumor level in treatment-naïve NSCLC. [18F]FDG dynamic PET-DCE MRI has the unique capability to capture the individual tumor biological behavior of NSCLC. Vascularity and perfusion properties are spatially variable in NSCLC [48, 49]. This wide variability has been recently highlighted in [18F]FDG PET compartmental analyses [50] and was qualitatively illustrated in our combined dynamic PET-DCE MRI study.

As expected, MRGlu and k_3_ PET microparameters were positively correlated in all the tumors, emphasizing the expected close relationship between the regional metabolic and phosphorylated rates of glucose. In more than half the tumors, both MRGlu and k_3_ were inversely correlated to K_trans_, v_p_, and v_b_, suggesting high metabolic but low perfused/vascularized cells, a well-known hallmark of tumor hypoxia or aggressiveness [20]. Recent head and neck ^18^F-FMISO [51] and preclinical VX-2 ^13^N-NH_3_ [52] PET/DCE MRI studies showed weak correlation between K_1_ and K_trans_ perfusion parameters. In our NSCLC [18F]FDG PET/DCE-MRI clinical study, the K_1_–K_trans_ correlations were also mainly weak. This general trend is not surprising considering the three following key concepts: First, perfusion reflects a weighted mixture of blood flow and permeability-surface area product [13, 53, 54] that depends, in the case of fixed flow and microvascular characteristics, on the tracer’s exchange properties—[18F]FDG is actively transported across the cellular membrane, whereas Gd is a purely extra cellular diffusive contrast agent. Second, the DCE Tofts models [46, 55] do not consider the intra-cellular space (ICS), whereas standard compartmental PET models [7] do not distinguish the extravascular extracellular space (EES) from the ICS, assuming steady state between EES and ICS at time of injection. Consequently, K_1_ depends on a mixed perfusion-extraction weighting of [18F]FDG that may, in the case of high metabolic rate conditions, overestimate the perfusion component [53].

Our study has several limitations. Our data sample was limited to 14 biopsy-proven NSCLC. Also, because pre-contrast T_1_-mapping was limited to 6 slices per tumor for practical considerations, we could not capture the multimodal correlations of the entire tumor volume. Compared to PET, DCE kinetic modeling showed higher voxel-wise fit errors. It is well-known that many factors hamper the accuracy of DCE pharmacokinetic modeling, making this approach highly challenging in clinical practice [56–58]. For illustration, analyzing the same patient and imaging data with multiple different commercially available software packages was reported to lead to within-patient variabilities of up to 74% in DCE-MRI measurements [59]. In our study, motion corruption was probably the major explanation of the measurement errors. The high temporal resolution of the DCE frames emphasized the motion corruption effects, which were only partially compensated by our standard motion correction method. For our study, the mean fraction of outliers used for the correlation analyses was under 10% among all the 14 tumors (7.8% ± 2.8%). A better availability of advanced motion compensation techniques [60] would be of particular interest. We did not include the K_i_ PET parameter but instead used the MRGlu parameter, which is the K_i_-glycaemia product normalized by the lumped constant (LC). We justified this choice because LC is arbitrarily set to 1 in oncology studies (the unknown true LC precludes any other value) [61, 62] making MRGlu a basic multiple of K_i_. A dual arterial input implementation has been recently proposed in few CT or MR-based perfusion studies [63–67], based on the fact that lung tumors may have a dual blood supply [68]. The selection of the correct model for the right tumor is limited by what is named the “mixed tissue conundrum” [69] and remains mainly driven by both its bias-variance tradeoff and clinical relevance. In this way, DCE Tofts models have become standards in oncology [70, 71] and have shown preclinical and clinical relevance in lung cancer specifically [15, 16, 72]. The dual AIF, however, has never been validated for dynamic PET analyses and therefore cannot be considered as a reference. Even though the majority of the included tumors were in the upper lobes, our results are prone to potential uncertainties related to respiratory motion artifacts and the uncertain efficacy of our motion compensation procedure. Finally, voxel-wise comparisons were performed on data resampled at 2 mm^3^ resolution and an 8-mm 3D Gaussian smoothing applied to the PET modality. The 8 mm Gaussian post-filtering was applied to the PET data to denoise the PET images and regularize the motion-corrupted time-varying activity curves. To some extent, neighboring voxels are expected to share similar behavior in a lesion of interest, and the smoothing process emphasizes this structural consistency at the regional intra-tumor level by reducing the granularity of noise in the data. Moreover, Gaussian kernels make the distribution of the data more reliable for further statistical analyses: for example, state-of-the-art multimodal neuroimaging analyses using statistical parametrical maps (SPM) typically use smoothing of the PET data before voxel-wise analyses, and wider smoothing kernels are frequently used. These pre-processing steps are not a problem for multimodal analyses of thin cortical structures at the voxel level (for example, see [73]). Despite the use of Gaussian smoothing on PET data, the 3D maps of both the PET and DCE kinetic parameters showed structured and consistent intra-tumor regional subparts, as illustrated in Fig. 6.

Despite these limitations, this study shows that simultaneous dynamic PET-MRI is feasible in NSCLC patients. This tends to demonstrate the potential application of simultaneous PET/MRI imaging to further characterize the individual biological tumor behavior in NSCLC in clinical practice. However, further studies are necessary to demonstrate the clinical utility of this approach.

## Conclusion

A dynamic “one-stop shop” procedure applied to NSCLC is technically feasible in clinical practice. Simultaneously acquired PET and DCE kinetic parameters assessed in a combined manner are not highly correlated in NSCLC, and these correlations showed a wide variability between tumors and patients. These results tend to suggest that PET and DCE kinetic parameters might provide complementary information for tumor characterization, and this might make PET-MRI a unique tool to characterize the individual tumor biological behavior in NSCLC.

## Supplementary information

**Additional file 1.** Voxel-wise Spearman correlation coefficients (r_s_) together with their respective bootstrap intervals (n = 1000 replications). Values in red correspond to statistically non-significant results.

## Data Availability

The datasets generated and analyzed during the current study are not publicly available, in accordance with the General Data Protection Regulation (GDPR) of the European Union, but are available from the corresponding author on request, after justification that meets the GDPR principles.
