# Supplementary figures and images for: 18F-FDG PET and DCE kinetic modeling and their correlations in primary NSCLC: first voxel-wise correlative analysis of human simultaneous [18F]FDG PET-MRI data
Source: EJNMMI Res. 2020 Jul 30;10:88. doi: 10.1186/s13550-020-00671-9 (PMC7392998; doi:10.1186/s13550-020-00671-9)

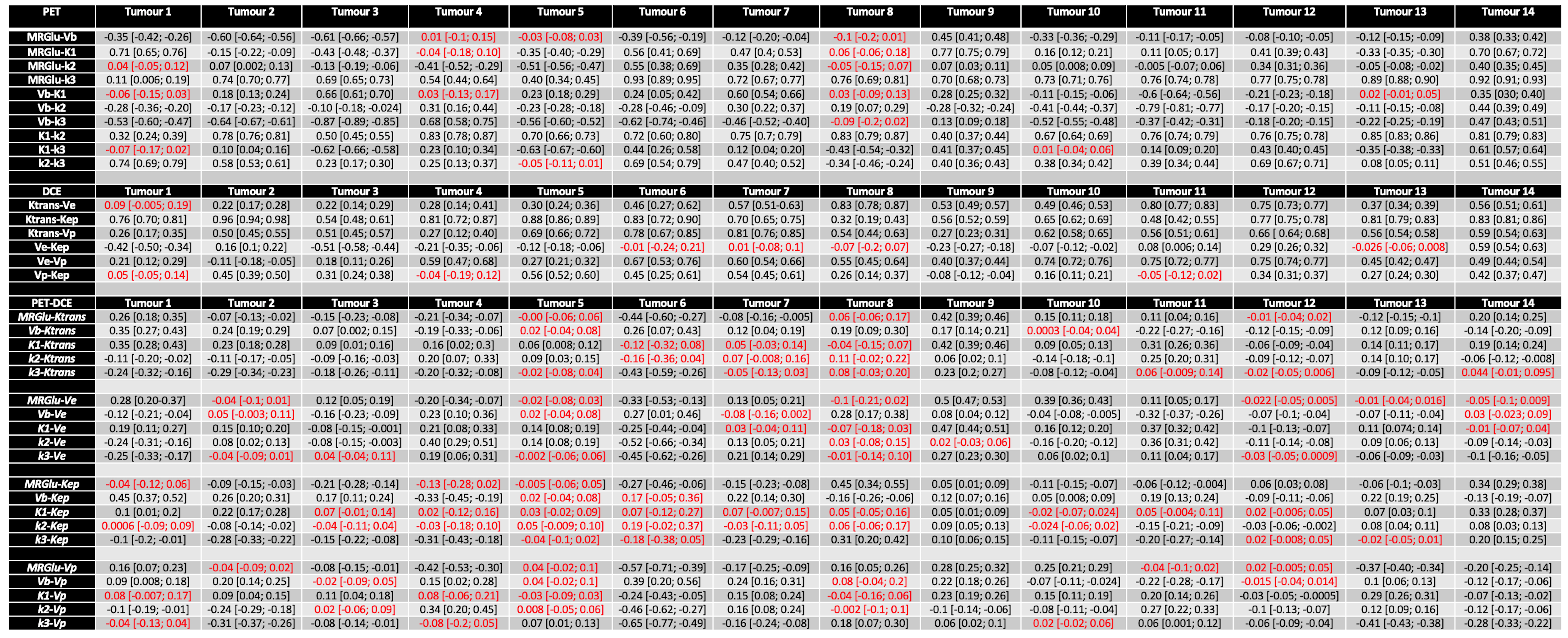

Supplement: Supplementary file 1 — Additional file 1. Voxel-wise Spearman correlation coefficients (rs) together with their respective bootstrap intervals (n = 1000 replications). Values in red correspond to statistically non-significant results. [file 13550_2020_671_MOESM1_ESM.png]
